# The exploration of iparomlimab and tuvonralimab combined with de−escalated chemotherapy as an innovative neoadjuvant treatment strategy for locally advanced cervical cancer: a case report from the NICE-CC trial

**DOI:** 10.3389/fimmu.2026.1801164

**Published:** 2026-04-24

**Authors:** Jie Lin, Haixin He, Bin Liu, Tongmei He, Xuemei Lei, Xinye Guo, Xingfa Chen, Xiang Zheng, Yang Sun

**Affiliations:** 1 Department of Gynecology, Clinical Oncology School of Fujian Medical University, Fujian Cancer Hospital, Fuzhou, China; 2 Department of Pathology, Clinical Oncology School of Fujian Medical University, Fujian Cancer Hospital, Fuzhou, China; 3 Department of Radiologic Diagnosis, Clinical Oncology School of Fujian Medical University, Fujian Cancer Hospital, Fuzhou, China

**Keywords:** case report, chemo-immunotherapy, complete response, de-escalated chemotherapy, iparomlimab and tuvonralimab, locally advanced cervical cancer, neoadjuvant

## Abstract

Recent research has explored the potential of using neoadjuvant dual immune checkpoint inhibitors (ICIs) combined with de−escalated chemotherapy in several locally advanced tumors to determine if such a combined regimen can enhance tumor response while minimizing toxicity. However, few related studies are focused on locally advanced cervical cancer (LACC). In this study, we present a case from the NICE-CC trial evaluating the feasibility of neoadjuvant dual immune checkpoint inhibitor (ICI) combined with a de-escalated chemotherapy regimen for LACC. A patient with stage IIB LACC had a high tumor burden and a presumed “immune cold” status, indicated by PD-L1 negativity with a Combined Positive Score (CPS) of 0. The patient achieved a pathological complete response (pCR) after receiving one cycle of neoadjuvant iparomlimab and tuvonralimab (simultaneously targeting PD-1 and CTLA-4) combined with standard chemotherapy, followed by two additional cycles of iparomlimab and tuvonralimab. Mechanically, the tumor microenvironment (TME) in this case was characterized by an abundance of tumor-infiltrating lymphocytes (TILs) and tertiary lymphoid structures (TLSs), which might be associated with improved responses to ICI therapy. In conclusion, this case highlights the potential of one cycle of neoadjuvant dual immunotherapy combined with standard chemotherapy, followed by two additional cycles of dual immunotherapy, for the treatment of LACC. This innovative treatment regimen warrants further investigation in the ongoing NICE-CC trial.

## Introduction

Cervical cancer is the fourth most common cancer among women in terms of both incidence and mortality (1). In 2022, there were approximately 660,000 new cases and 350,000 deaths worldwide (1), placing a significant burden on healthcare systems and patients’ families. Alarmingly, up to 37% of cervical cancer cases are classified as locally advanced, with even higher rates observed in limited-resource regions, where the lack of effective screening programs and essential medical equipment complicates treatment strategies (2, 3). Even in developed countries like the United States, locally advanced cervical cancer (LACC) presents a significant treatment challenge, with a 5-year survival rate of 60%, despite the use of modern chemoradiation (CRT) techniques (4).

The mainstream treatment for LACC is concurrent chemoradiotherapy (CCRT) and brachytherapy (5). However, platinum-based neoadjuvant chemotherapy (NACT) followed by radical hysterectomy could be an attractive alternative when timely CCRT is unavailable, especially in low-resource areas. Such a recommendation is based on the results from several randomized clinical trials, which indicate that overall survival (OS) is non-inferior, despite a poorer progression-free survival (PFS) (6, 7). Recent pioneering research suggests that adding anti-programmed cell death 1 (PD-1) inhibitors to the standard chemotherapy in neoadjuvant settings yields a higher pathological complete response (pCR) rate, ranging from 35% to 66.7% (8–10), compared to approximately 17% pCR with chemotherapy alone in LACC (11, 12). Such a novel combination significantly outperforms the standard neoadjuvant chemotherapy and dramatically minimizes the need for CCRT or postoperative radiotherapy in patients with LACC. For instance, in cervical cancer with stage IB3/IIA2, the addition of three cycles of tislelizumab (anti-PD-1) to standard neoadjuvant chemotherapy results in a pCR rate of up to 66.7%. Additionally, only 7 out of 30 patients (23.3%) require adjuvant therapy after neoadjuvant treatment (10). Moreover, the COLIBRI trial further supports the use of immunotherapy in the neoadjuvant setting, demonstrating the efficacy and safety of inivolumab combined with ipilimumab as induction therapy, followed by chemoradiation therapy (CRT) in LACC patients, achieving an overall response rate (ORR) of 97.5% (13). Moving forward, a new era is emerging, where we are witnessing more promising and effective immunotherapy drugs, along with innovative treatment combinations that have fewer adverse events (AEs).

Recent research set the favorable signals that a combination regimen of dual blockade immunotherapy with reduced/eliminated chemotherapy could enhance treatment efficacy while minimizing toxicity in neoadjuvant settings for several solid tumors, including colon cancer (14), oral cavity squamous cell carcinoma (15), and head and neck cancers (16), regardless of PD-L1 expression. Findings from the NICHE-2 study, which involved patients with locally advanced mismatch repair-deficient (dMMR) colon cancer treated with neoadjuvant nivolumab and ipilimumab, demonstrated an impressive pathological response rate of 98% among 111 patients, with a pCR rate as high as 68% (17). An interesting clinical question arises: whether a de−escalated chemotherapy regimen combined with dual immunotherapy could also be effective and simultaneously reduce chemotoxicity in neoadjuvant settings for LACC. However, there has been limited research focused on such innovative reform in neoadjuvant chemo-immunotherapy specifically for cervical cancer.

To address this gap, our study team initiated a multicenter, open-label phase II trial called “Neoadjuvant therapy of iparomlimab and tuvonralimab combined with chemotherapy-extenuated for locally advanced cervical cancer (NICE-CC trial)” (18) (ClinicalTrials.gov Identifier: NCT07055399). Iparomlimab and tuvonralimab is a novel single bifunctional MabPair product targeting PD-1 and cytotoxic T lymphocyte-associated protein-4 (CTLA-4) developed by Qilu Pharmaceutical Co., Ltd in China (19), which is expected to improve tumor response due to synergistic activity. Eligible patients are enrolled in the NICE-CC Trial and receive one cycle of neoadjuvant iparomlimab and tuvonralimab in combination with standard chemotherapy, followed by two additional cycles of iparomlimab and tuvonralimab, then continued with radical surgery. The NICE-CC trial aims to investigate whether this innovative combination can maintain efficacy while minimizing toxicity, as well as reducing the need for postoperative radiotherapy or chemoradiotherapy. Here, we present a high-tumor-burden pCR case in the NICE-CC trial, who was staged as IIB and PD-L1-negative (Combined Positive Score, CPS = 0), providing a preliminary signal of efficacy and safety in the neoadjuvant setting for LACC.

## Case presentation

**Figure 1:**
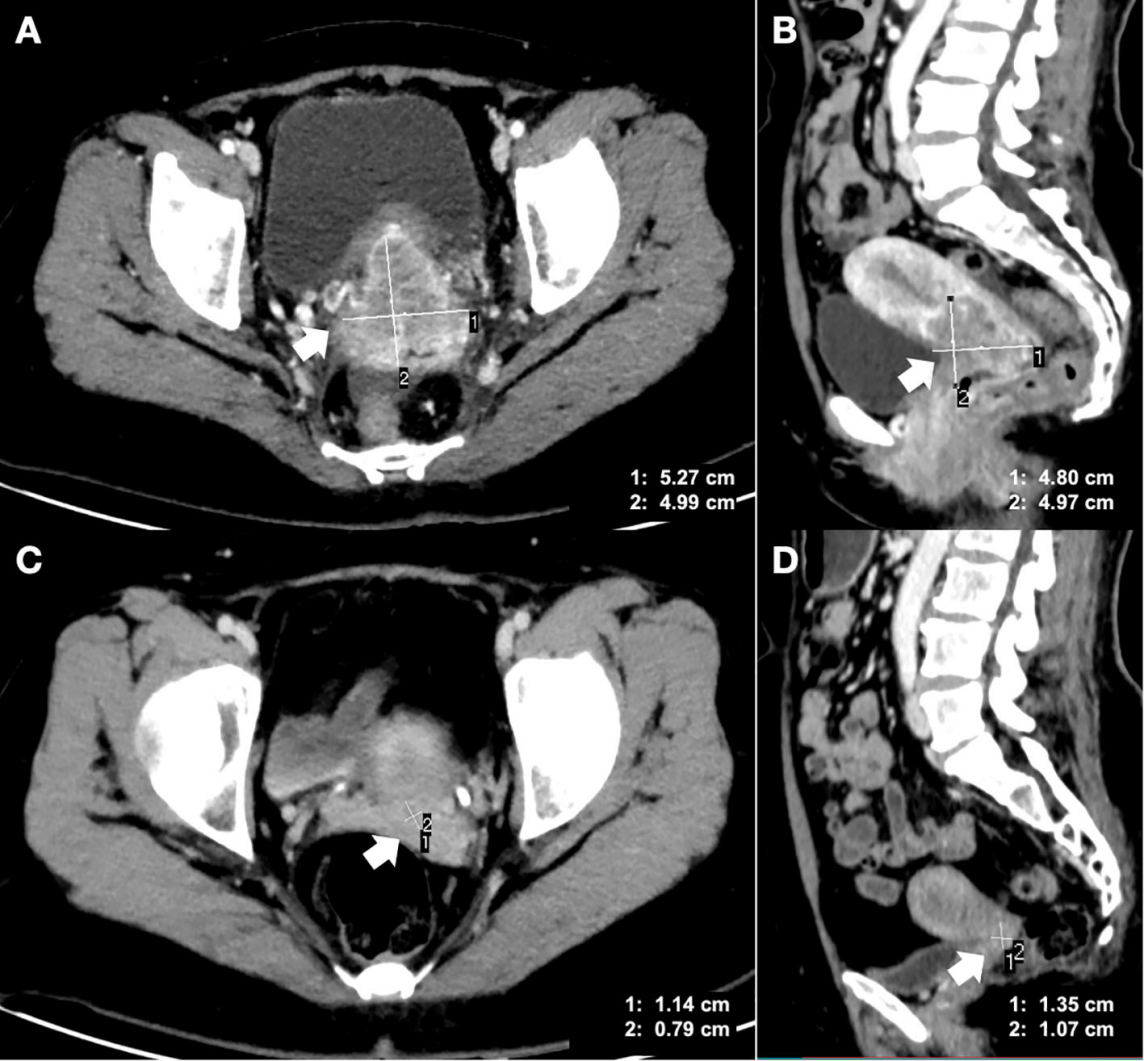
Comparison of enhanced CT images at pre-treatment (2025.09.04) and pre-surgery (2025.11.24). **(A)** Tumor mass in the cervix at the transverse position (5.27 cm×4.99 cm) prior to treatment. **(B)** Tumor mass in the cervix at the sagittal position (4.80 cm×4.97cm) prior to treatment. **(C)** Tumor mass in the cervix at the transverse position (1.14 cm×0.79 cm) prior to surgery. **(D)** Tumor mass in the cervix at the sagittal position (1.35 cm×1.07 cm) prior to surgery. In each subfigure, the white arrow points to the tumor mass. CT, computed tomography.

A 49-year-old woman with regular menstrual cycles was admitted to Fujian Cancer Hospital, Fuzhou, China, on September 5, 2025, due to one month of irregular vaginal bleeding. She was diagnosed with grade 2 cervical squamous carcinoma, with positive HPV types 16 and 53. Immunohistochemistry (IHC) confirmed negative PD-L1 (clone 28-8; Abcam, Cambridge, UK) with a CPS of 0, Ki-67 (90%), and P16 (+). A contrast-enhanced computed tomography (CT) scan revealed a cervical mass measuring 5.27 cm by 4.99 cm, with tumor infiltration into the left parametrium and the vaginal vault (**Figures 1A, B**). Additionally, her serum SCC level was significantly elevated at 30.3 ng/ml, far exceeding the normal average of 1.5 ng/ml, indicating a heavy tumor burden. She was classified as stage IIB according to the International Federation of Gynecology and Obstetrics (FIGO) 2018 staging system.

The patient was quite concerned about the side effects of concurrent chemoradiotherapy and decided to participate in the NICE-CC trial. She met the inclusion criteria and received three cycles of neoadjuvant treatment. During the first cycle, she was administered iparomlimab and tuvonralimab (5 mg/kg intravenously), nab-paclitaxel (260 mg/m² intravenously), and carboplatin (AUC = 5, intravenously). For the following two cycles, she received only iparomlimab and tuvonralimab (5 mg/kg intravenously) at three-week intervals. The Common Terminology Criteria for Adverse Events (V.5.0) is used to evaluate AEs after the first dose of treatment drugs to 30 days after the last dose. During treatment, the patient experienced Grade 1 muscle soreness on the first day of each treatment cycle with iparomlimab and tuvonralimab. Additionally, the patient developed a Grade 1 rash on the second day of the second cycle of iparomlimab and tuvonralimab, which lasted for one week. This rash resolved with the use of a corticosteroid ointment.

**Figure 2:**
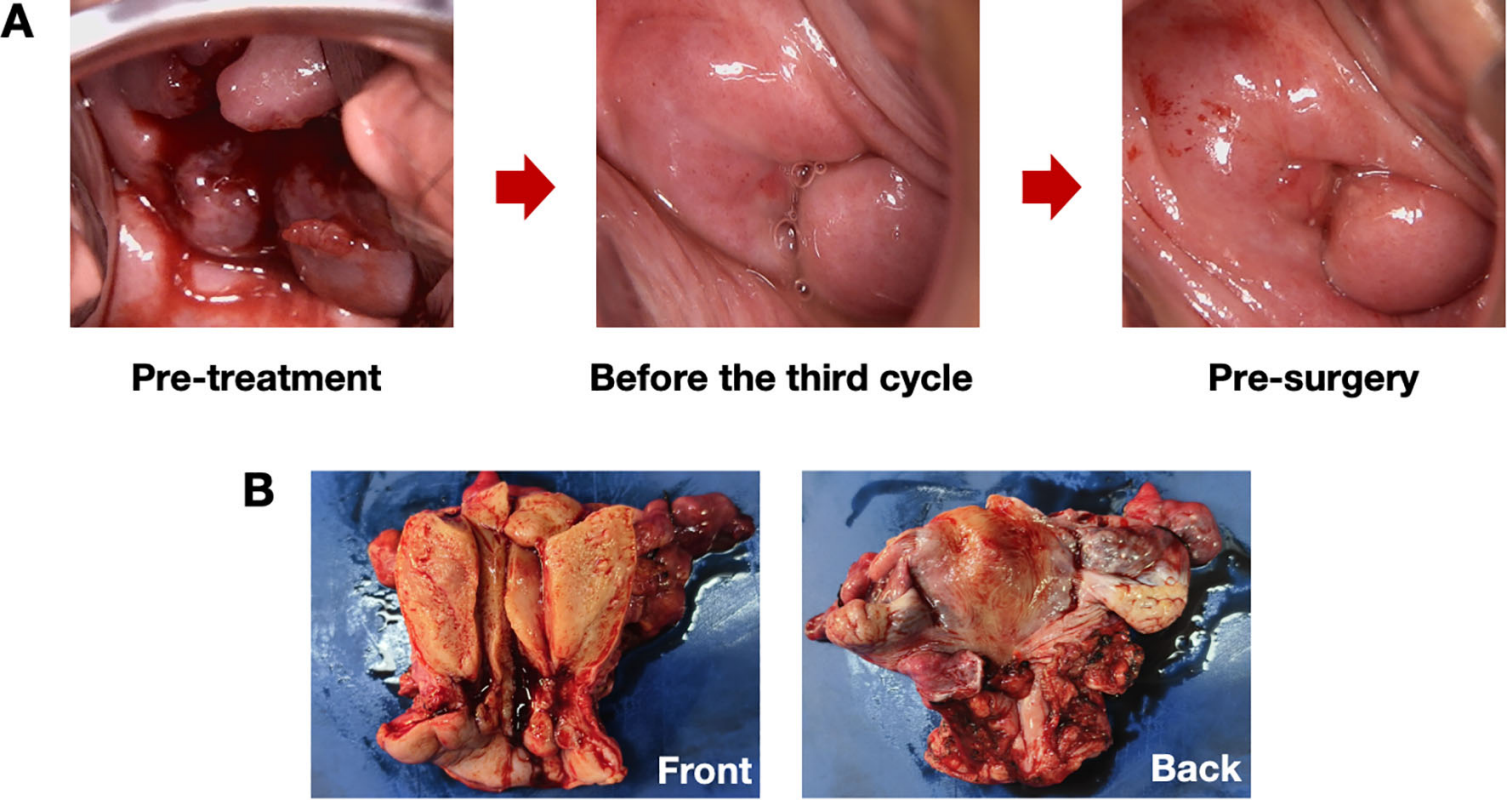
The morphology of the tumor changed during treatment. **(A)** The colposcopy findings for the patient during treatment are as follows: Before treatment, the cervix exhibited a mass resembling a “volcano,” measuring 5.0 cm in diameter, with easy hemorrhage and a tough texture. After three cycles of neoadjuvant treatment, the tumor had shrunk to the point of being undetectable. The texture of the cervix was soft, and its color was pinkish-white. **(B)** A gross pathological examination after surgery, viewed from both the front and back, showed no visible tumor in the cervix.

After that, a contrast-enhanced CT scan was conducted, revealing a tumor that was nearly unmeasurable at 1.14 cm x 0.79 cm (**Figures 1C, D**). The colposcopy also indicated that the tumor lesion had disappeared (**Figure 2A**). Furthermore, laboratory tests showed that the serum SCC level had rapidly decreased to a normal range of 0.8 ng/ml. All the data suggested that the patient responded well to this innovative neoadjuvant regimen. Based on a comparison of the pre-treatment and pre-surgery CT images, the clinical tumor response was assessed as a partial response (PR) according to the Response Evaluation Criteria in Solid Tumors (RECIST) 1.1 criteria (20). On November 26, 2025 (almost 4 weeks after the last dose of treatment), the patient underwent a three-hour open radical surgery (**Figure 2B**), which included a radical hysterectomy, oophorectomy, pelvic lymphadenectomy, and para-aortic lymphadenectomy, with a blood loss of 100 ml. The patient experienced only Grade 1 anemia, with a postoperative hemoglobin level of 92g/L. There were no other surgical complications, such as bleeding, infection, or bowel obstruction, and no AEs were recorded within 30 days after the procedure. Moreover, no immune−related AEs were observed in thyroid, hepatic, gastrointestinal, or pulmonary symptoms.

**Figure 3:**
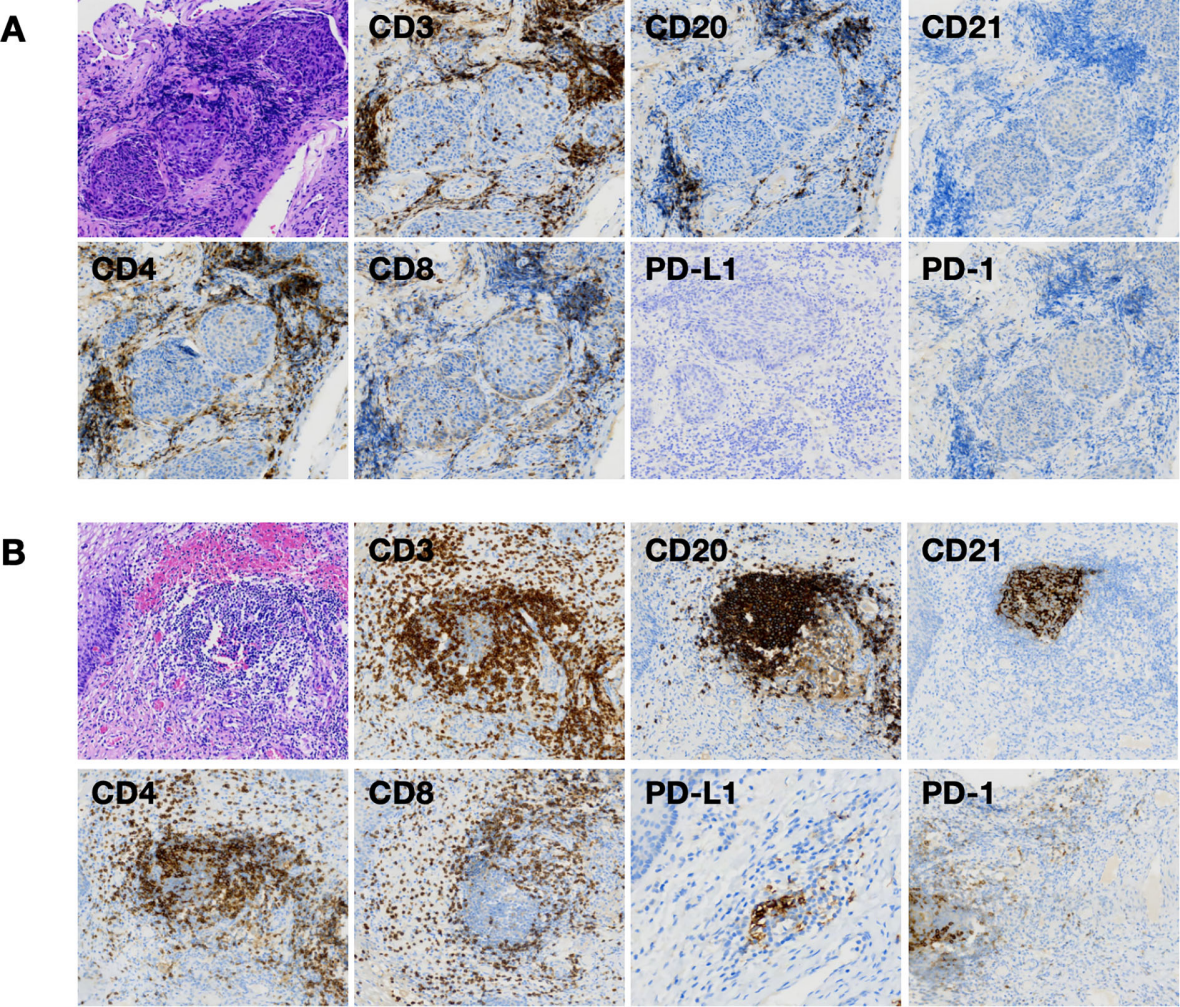
Comparison of the morphological histopathology of tumor tissue in the cervix **(A)** and the area of the cervix where the tumor was located after three cycles of neoadjuvant treatment **(B)**. **(A)** H&E staining revealed the malignant behavior of the tumor cells and enriched TILs. An IHC panel that includes CD3, CD20, CD21, CD4, and CD8 further indicates an enrichment of T cells and B cells surrounding the tumor tissue at baseline. **(B)** H&E staining shows no evidence of malignant cells after three cycles of neoadjuvant treatment. The presence of TLSs is confirmed by the IHC panel. IHC was used to label CD3^+^ T cells, CD20^+^ B cells, CD21^+^ follicular dendritic cells, CD4^+^ T cells, and CD8^+^ T cells. The presence and maturity of TLSs were assessed using immunohistochemical markers (CD3, CD20, and CD21); CD4^+^ and CD8^+^ T cells represent T cells of the helper/inducer phenotype and T cells of the suppressor/cytotoxic phenotype, respectively; H&E staining × 100; IHC × 100. H&E: hematoxylin and eosin stain, IHC: immunohistochemistry; TLSs: tertiary lymphoid structures; TIL:tumor-infiltrating lymphocytes.

Unexpectedly, the patient reached a pCR with this innovative treatment regimen, which had not been previously investigated in cervical cancer. Two expert pathologists specializing in gynecologic cancers will review the histological examination of the entire resected specimen in a blinded fashion. pCR is defined as the absence of viable tumor cells across all slides. No cancer cells were found in the cervix, uterus, ovaries, or vagina. Lymph-vascular space invasion was also absent. A total of 3 aortic and 22 pelvic lymph nodes were removed, and no positive lymph nodes were detected. IHC analysis further revealed an enrichment of tumor-infiltrating lymphocytes (TILs). The density of lymphocytes expressing CD3, CD4, CD8, CD20, and CD21 was significantly high around the tumor tissues at baseline (**Figure 3A**). After three cycles of neoadjuvant treatment, the presence and maturation of tertiary lymphoid structures (TLSs) were observed, indicating a strong association between TLSs and a favorable immune response (**Figure 3B**). Additionally, an elevated level of PD-1 expression was detected, suggesting it could serve as a signal for an enhanced immune response. Based on the pathological results, the patient did not require any adjuvant therapy, in accordance with the guidelines set by the National Comprehensive Cancer Network (NCCN) guidelines (5). Post-operative surveillance was conducted every three months in the outpatient clinic. During the most recent 3-month visit, the patient remained asymptomatic. The pelvic examination was unremarkable, and imaging showed no evidence of local recurrence or distant metastasis. Tumor markers stayed within the normal range. This case suggests that even cervical cancer patients with PD-L1-negative expression (CPS = 0) could benefit from the bifunctional ICI combination and achieve pCR, even with reduced chemotherapy.

## Discussion

We reported a pCR in a heavy tumor burden LACC case staged FIGO IIB with negative PD-L1 expression after receiving one cycle of neoadjuvant iparomlimab and tuvonralimab combined with standard chemotherapy, followed by two additional cycles of iparomlimab and tuvonralimab. This case provides a preliminary “signal” of efficacy for this neoadjuvant combination regimen in LACC. With this novel attempt, the patient avoided the chemoradiotherapy or postoperative radiotherapy, as well as radiation-caused side effects, such as diarrhea, cystitis, and proctitis. Additionally, the presence of TILs and TLSs could be promising indicators for predicting the effectiveness of ICI therapy. However, it brings concern to us that radiological images may underestimate the early response to ICIs, which aligns with findings in breast cancer (21)and melanoma (22), warranting further investigation.

**Figure 4:**
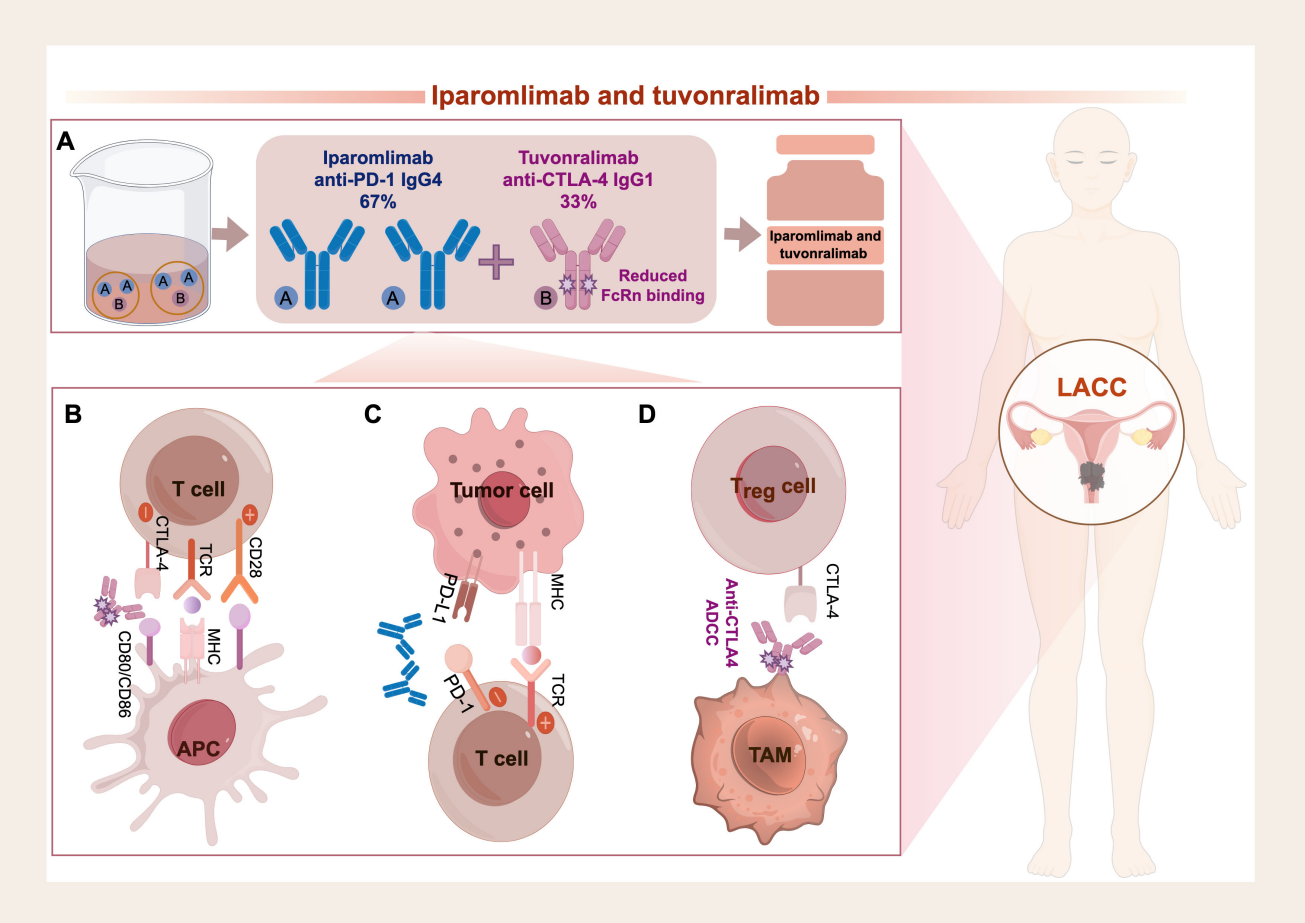
The function of iparomlimab and tuvonralimab **(A)** The composition and structure of Iparomlimab and Tuvonralimab. **(B)** Tuvonralimab enhances early T cells by preventing CTLA-4 from inhibiting CD28-mediated co-stimulation. **(C)** Iparomlimab reverses the exhaustion of effector T cells within tumors, restoring their cytotoxic activity and cytokine production by blocking the PD-1/PD-L1 pathway. By targeting both pathways, cancer cells are prevented from escaping the effects of exhausted T-cell immunity while also activating T cells during the early stages of the immune response, leading to more effective tumor clearance. **(D)** Tuvonralimab has the potential to deplete Tregs within the tumor microenvironment by blocking the CTLA-4 pathway. Additionally, the unique design of the Fc region of tuvonralimab could trigger ADCC, which could recruit immune cells to help eliminate the Tregs. (The figure was drawn by Figdraw (https://www.figdraw.com/). CTLA-4, cytotoxic T lymphocyte-associated protein 4; PD-1, programmed cell death 1; PD-L1, programmed cell death ligand 1; ADCC, antibody-dependent cellular cytotoxicity; Tregs, regulatory T cells.

Recent investigations into neoadjuvant chemo-immunotherapy studies for LACC have primarily focused on a single immune checkpoint, particularly in PD-L1-positive populations, with pCR rates of 35%∼66.7% and≧3AEs of 26.7%∼40% (8–10). This has led to an inspiration of whether dual ICIs therapy could be more effective than single-agent therapy, as well as benefit PD-L1-negative populations. Several clinical trials have demonstrated that dual ICI therapy is more effective and reasonably safe compared to single-agent treatments for various solid tumors, including colon cancer (23), melanoma (24), and glioblastoma (25). Iparomlimab and tuvonralimab is a bifunctional combination produced via the MabPair technology platform. This single product consists of two engineered monoclonal antibodies: an anti-PD-1 IgG4 antibody and an anti-CTLA-4 IgG1 antibody, which are expressed in a fixed approximate ratio of 2:1 from a single cell line (26) (**Figure 4A**). In the present study, a pCR was achieved in a cervical cancer patient with a significant tumor burden, suggesting that Iparomlimab and Tuvonralimab may have a promising anti-tumor efficacy and a favorable safety profile in the treatment of cervical cancer, which needs further investigation in the NICE-CC trial.

Iparomlimab and tuvonralimab specifically targets and binds to PD-1 and CTLA-4 simultaneously, effectively blocking the PD-1/PD-L1 and CTLA-4/B7 signaling pathways (27) (**Figures 4B, C**). By inhibiting both targets, the suppression of T lymphocytes is reversed, thereby restoring their immune function (28), which enables a more thorough clearance of tumors (29, 30). Moreover, dual blockade promotes the formation of long-lived memory CD8^+^T cells, which help prevent tumor relapse by maintaining antitumor immune surveillance (31). It is important to note that tuvonralimab has the potential to deplete regulatory T cells (Tregs) within the TME by blocking CTLA-4. Additionally, the unique design of the Fc region of tuvonralimab could trigger antibody-dependent cellular cytotoxicity (ADCC), which could recruit immune cells to help eliminate the Tregs (19, 26, 27) (**Figure 4D**).

Recently, overtreatment prevention has become an increasingly important consideration due to the high number of patients suffering unnecessary increased toxicity with more intense and prolonged therapy (21). Motivated by the concern that traditional chemotherapy may impair T-cell activity, potentially diminishing the immune response when used in conjunction with ICIs (16, 21, 32), we initially evaluated a chemotherapy-free regimen for second- or later-line treatment (33)and a de-escalated chemotherapy regimen for first-line treatment (34) in patients with persistent, recurrent, or metastatic advanced cervical cancer. Our study yielded promising results, reporting a median PFS of 9.4 months for second- or later-line treatment, as well as a median PFS of 12.48 months for first-line treatment, which aligns closely with that reported in KEYNOTE-826 (35). Both trials demonstrated the feasibility of reducing the number of conventional chemotherapy cycles in the first and later-line treatment of advanced cervical cancer. While cross-tumor learning can provide valuable insights, there is currently no direct evidence supporting the use of chemo-less or chemo-reduced treatment in the neoadjuvant setting for cervical cancer. Therefore, we launched the NICE-CC trial to evaluate the feasibility of reduced-cycle chemotherapy regimens in the neoadjuvant setting for LACC (18). The successful attempt with the pCR case offers a promising outlook on this novel neoadjuvant combination regimen, which requires further investigation in the ongoing NICE-CC trial.

Surgery after ineffective NACT could potentially delay the timing of optimal interventions (radiotherapy) and result in unfavorable survival outcomes. Given this consideration, in the NICE-CC trial, we recommend prompt initiation of CCRT and brachytherapy for patients who do not respond to neoadjuvant immunochemotherapy. Such a recommendation is also based on the reason that even after radical surgery, these patients will still require various treatment strategies, which can result in longer treatment durations, increased costs, and more severe side effects. More importantly, rescue surgery could be reserved as a last option for SD patients if they do not respond to radiotherapy. Hence, identifying the underlying mechanisms of action and detecting biomarkers to define the immunosensitive population is a priority, as it may help avoid ineffective neoadjuvant chemo-immunotherapy.

Tumor-infiltrating lymphocytes (TILs) are key effectors in the fight against cancer within TME. They play a crucial role in shaping the local immune microenvironment, orchestrating the anti-tumor immune response, and influencing immunotherapy outcomes (36, 37). At baseline, TIL enrichment is observed around the tumor in this case, and TLSs have formed after neoadjuvant immunotherapy. TLSs are ectopic lymphoid aggregates that contain B cells, T cell zones, high endothelial venules, and antigen-presenting cells (38). These structures typically form in or near tumors and other chronically inflamed tissues, playing vital roles in local antigen presentation, lymphocyte priming, and the coordination of adaptive immunity (39, 40). The presence of TLSs within the TME is associated with improved responses to ICI therapy, making them valuable biomarkers for predicting the effectiveness of ICI therapy across various cancers (40, 41). A systematic review analyzed 17 studies involving 4,291 lung cancer patients and found that those with high levels of TLS or positive TLS (TLS/TLS^+^) experienced better OS (hazard ratio [HR] = 0.66, 95% confidence interval [CI]: 0.50–0.88) and disease-free survival (DFS) (HR = 0.46, 95% CI: 0.33–0.64). Subgroup analysis indicated that among patients receiving neoadjuvant chemoimmunotherapy, high TLS/TLS^+^ levels were associated with prolonged DFS (HR = 0.21, 95% CI: 0.05–0.93) (42).

Promoting TLS maturation is a potential strategy to improve antitumor responses and immunotherapy outcomes (43, 44). For instance, the Tang team (45) has identified two types of immature TLSs through near-single-cell spatial transcriptomic mapping in hepatocellular carcinoma (HCC). The first type, called conforming TLSs, appears to have a positive effect on immunotherapy responses. In contrast, the second type, referred to as deviating TLSs, does not respond effectively to immunotherapy. Further research has shown that a metabolic microenvironment enriched with tryptophan contributes to the impaired maturation of these TLSs. Inhibiting tryptophan metabolism has been found to promote the maturation of intratumoral TLS and enhance tumor control, working synergistically with anti-PD-1 treatments (45). Based on the findings from this case, our study team is now investigating the underlying mechanism by which dual ICIs remodel TLS to promote pCR in LACC patients.

We proposed a novel treatment strategy combining dual ICIs with reduced chemotherapy, demonstrating promising anticancer effects. Our study, however, did have several limitations. First, we present only one CR case, as the NICE-CC trial has just been initiated, which prevents us from deeply investigating the immunological mechanisms underlying the CR status. Second, it is preferable to evaluate tumor response using MRI. However, we utilized enhanced CT instead due to the patient’s financial considerations. Third, whether patients with pCR could achieve better long-term survival requires further follow-up. Forth, large samples and longer follow-up data are awaited to support the efficacy of this novel reform neoadjuvant regimen. Nevertheless, this case report will still provide valuable information for the future application of iparomlimab and tuvonralimab in the neoadjuvant setting for LACC.

## Conclusion

This CR case report highlights the potential benefits of a novel neoadjuvant treatment regimen that includes one cycle of iparomlimab and tuvonralimab combined with standard chemotherapy, followed by two additional cycles of iparomlimab and tuvonralimab. The efficacy and safety of this treatment warrant further investigation in the ongoing NICE-CC trial, along with an exploration of the underlying mechanisms.

## Data Availability

The raw data supporting the conclusions of this article will be made available by the authors, without undue reservation.
